# The relationship between hair cortisol and trauma sequelae in motor vehicle crash survivors: the role of childhood trauma experiences

**DOI:** 10.1038/s41398-025-03295-6

**Published:** 2025-03-19

**Authors:** Ileana Schmalbach, Susann Steudte-Schmiedgen, Vanessa Renner, Philipp Drees, Katja Petrowski

**Affiliations:** 1 Department of Medical Psychology and Medical Sociology, University Medical Centre of the Johannes Gutenberg-University Mainz, Mainz, Germany; 2 Department of Psychotherapy and Psychosomatic Medicine, Faculty of Medicine and University Hospital Carl Gustav Carus, TUD Dresden University of Technology, Dresden, Germany; 3 University Hospital of the University Johannes-Gutenberg Mainz, Department for Orthopedics and Trauma Surgery, Mainz, Germany; 4 Faculty of Medicine and University Hospital Carl Gustav Carus, TUD Dresden University of Technology, Dresden, Germany

**Keywords:** Predictive markers, Prognostic markers

## Abstract

Previous research highlights inconsistent associations between premorbid hair cortisol concentrations (HCC) and posttraumatic stress disorder (PTSD) symptoms, often neglecting the critical role of childhood trauma (CT) in civilian populations. To address this gap, our study investigates the predictive value of HCC for PTSD symptoms following a motor vehicle crash (MVC), extending our prior findings by assessing CT as a moderator within a sample that includes participants with and without CT. We hypothesize that pre-MVC HCC is positively associated with PTSD risk and that this relationship is moderated by early adversity. We examined *N* = 272 participants with a traumatic brain injury aged 18–65 years who experienced a MVC between 2010 and 2020. Cortisol concentrations were determined in 3 cm scalp-near segments of hair samples that were obtained at the emergency room shortly after the MVC (t1). Participants completed measuring instruments capturing symptoms of posttraumatic stress (Posttraumatic Diagnostic Scale [PDS]; Impact of Event Scale-Revised [IES-R]) and Childhood Trauma Questionnaire (CTQ). PDS and IES-R were re-collected three months post-MVC (t2). Elevated pre-MVC HCC predicted PTSD symptoms (*p* < 0.05), emphasizing the role of chronic stress and HPA axis dysregulation in PTSD. Contrary to our hypothesis, CT did not moderate this relationship, suggesting that HCC’s impact on PTSD is independent of early adverse experiences. In this context, CT emerged as an independent predictor of PTSD at the 3-month follow-up, underscoring its lasting influence on psychological trauma vulnerability, particular in the face of recent adversity. Our study confirmed that elevated pre-MVC HCC levels predict PTSD symptoms. Although childhood trauma did not moderate this relationship, it independently predicted PTSD at follow-up. These findings underscore the lasting impact of early adversity on mental health, highlighting the importance of considering both HPA axis regulation and trauma history to develop targeted interventions for adults exposed to new stressors.

## Background

Throughout life, individuals face stressors that may lead to post-traumatic stress disorder (PTSD), with early adversity, such as childhood trauma (CT), significantly increasing the risk of later psychopathology [1]. Early adversity is thought to have long-term effects on the hypothalamus-pituitary-adrenal (HPA) axis, leading to persistent cortisol dysregulation and a heightened risk of PTSD following future trauma [2–4]. In this regard, a recent meta-analysis [5] revealed a strong relationship between childhood trauma and PTSD symptoms in adulthood. However, the exact relationship remains unclear. While some studies support that early adversity leads to lasting HPA axis disruption, contributing to poor health outcomes later in life [6], others challenge this view, showing inconsistent cortisol changes even in the face of new adversity, thus questioning the HPA axis’s role in post-adversity health [7]. These mixed findings suggest the relationship between stress responses and mental health may be more complex, with early adversity potentially moderating how stress impacts long-term psychological outcomes [8–10].

In light of past evidence, hair cortisol concentrations (HCC) allow a view on long-term cortisol secretion [11, 12], potentially delivering insight regarding HPA dysregulation in PTSD. The HPA axis is central to the pathophysiology of PTSD [13], as it governs the body’s stress response. Acute stress triggers cortisol secretion, which helps mobilize energy to cope with stressors, with cortisol levels typically downregulating over time to restore balance. However, chronic stress can dysregulate cortisol secretion, either through hyperactivity or hypoactivity of the HPA axis [14, 15], fostering PTSD development [15, 16].

Building on this understanding of the HPA axis’s role in PTSD, past evidence reflects ambiguous results. On the one hand, a systematic review by Schindler-Gmelch et al. [17] highlighted that lower baseline hair cortisol concentrations (HCC) could serve as a predictor for PTSD symptoms following new trauma exposure, suggesting a potential prognostic utility of HCC. Nonetheless, prospective studies remain limited, and the evidence regarding HCC’s predictive value for PTSD symptomatology is still inconclusive, with studies offering conflicting results. While some findings [18] revealed that decreased HCC may predict PTSD symptoms, Pacella et al. [19] identified elevated HCC as a predictor of PTSD symptom onset following trauma. Even so, their hair samples were not collected immediately after the traumatic event possibly mirroring peritraumatic HCC. Our recent findings [20] contribute to this discourse by demonstrating that increased pre-traumatic HCC can predict avoidance behaviors in individuals with a first-time and single trauma exposure. However, this relationship did not extend to other trauma-related symptoms such as intrusion or hyperarousal. On the other hand, Sopp et al. [21] found no predictive value of baseline HCC for PTSD symptoms, particularly when new trauma exposure was not considered. These discrepancies may be attributed to methodological differences, such as the timing of sample collection, as well as variations in study populations (e. g., non-civilian samples, trauma types). Furthermore, the impact of early adversity on HPA axis functioning, was often not accounted for in the reported studies, potentially influencing the observed outcomes.

In sum, previous research reveals an inconsistent relationship between premorbid HCC and PTSD, often neglecting the pivotal role of early adversity, thereby limiting the clarity of existing findings. Moreover, there is a notable scarcity of longitudinal studies assessing premorbid HCC and trauma outcomes in civilian populations facing recent adversity. To address this gap, our study investigates the predictive value of integrated HCC for PTSD symptoms in individuals who experienced a motor vehicle crash (MVC), as a recent adversity. We build on our prior research on pre-MVC HCC and PTSD symptoms [18] by incorporating CT as a moderator. Our earlier study [20] analyzed pre-MVC HCC in a trauma-naïve sample. Considering CT’s known impact on HPA axis functioning, we hypothesize that pre-MVC HCC is positively associated with PTSD risk, with this relationship moderated by early adversity.

## Method

### Participants and procedure

The current investigation is part of a comprehensive research initiative exploring various outcomes among traffic accident victims suffering from central nervous system injuries. The study cohort consists of individuals diagnosed with traumatic brain injury (TBI) resulting from motor vehicle crash (MVCs), who underwent inpatient treatment at a trauma and reconstructive surgery clinic and polyclinic in Dresden and Mainz, Germany, spanning from 2010–2020. Patients aged 18–65 years were contacted within the first two days following the MVC. Eligibility criteria were established through an initial review of medical records, considering factors such as consciousness, chronic somatic conditions, and medication usage. Participants meeting eligibility criteria and able to provide written consent, as well as willing to furnish hair samples (min. hair length of 3 cm), were briefed on the study objectives. Exclusion criteria included smoking more than ten cigarettes daily, recent use of psychotropic or corticosteroid medications, chronic (e.g., cancer, diabetes, rheum, HIV, heart diseases) or acute illnesses (e. g., bacterial infections), pregnancy, engagement in shift work (night shifts, alternating schedules), and mental disorders based on structured interviews (SCID; Structured Clinical Interview for DSM-IV Disorders; [22]). Participants with a history of previous trauma were included. The final sample comprised *N* = 272 participants. Data were collected at two time points: Hair samples were collected within the two days (t1) at the hospital. Psychometric data were collected within 10 days after an individual appointment with the participants (t1) and three months post-MVC (t2). The regional ethics committee of the Medical Faculty at the Technical University of Dresden approved the study (Ethics No. EK 65022010).

### Measurement instruments

The *Childhood Trauma Questionnaire* (CTQ; [23]) was implemented to determine childhood adversities covering five domains: emotional abuse, physical abuse, emotional neglect, and physical neglect. Respondents rated 28 items on a Likert scale ranging from 1 (not at all) to 5 (very often). Trauma severity was categorized based on Häuser et al. [24] as follows: 25–34 (mild), 35–39 (moderate), and 40–125 (severe). The CTQ demonstrates strong psychometric properties, with internal consistency coefficients ranging from α = 0.89–0.95 [25, 26].

The *Impact of Event Scale* – revised (IES-R; [27, 28]) was implemented to assess the severity of PTSD within the past 7 days in a hospital setting. The IES-R measure three core symptom clusters based on 22 items: Intrusion, Avoidance, and Hyperarousal. The scale ranges from 0 (not at all) to 4 (extremely). A total IES-R score of 33 or over 88 indicates the likely presence of PTSD. The internal consistency of the questionnaire is *α* = 0.96 and the convergent validity with the PTSD Checklist (PCL-5; *rho* = 0.84). With a cut-off of 50, the IES-R demonstrates a sensitivity of 0.91 and specificity of 0.82 [29].

The *Posttraumatic Diagnostic Scale* (PDS; [30]) conveys 49 Items and was employed for additional diagnostic value for PTSD assessment based on DSM-IV criteria, experienced in the past 30 days. The scale begins with a checklist of potentially traumatic events. The specific scale items can be rated on a 4-point Likert scale from 0 (“not at all or only one time”) to 3 (“five or more times a week/ almost always”). Symptom severity scores range from 0 to 51, classified as follows: 0 (No symptoms), 1–10 (Mild), 11–20 (Moderate), 21–35 (Moderate to severe), and 36+ (Severe symptoms). The scale possesses good psychometric properties (*α* = 0.91, retest reliability *rho* = 0.84, and convergent validity with DSM-5 Checklist [PCL-5] *rho* = 0.91 [31].

### Hair cortisol analysis

In the present study a single hair segment was analyzed per sample (pre-MVC HCC_t1_). The hair samples were immediately obtained at the emergency room directly from the occipital scalp using 2–3 mm diameter patches of hair, which were subsequently trimmed to 3 cm segments. Hair samples were wrapped in aluminum foil and sent to our laboratory for analysis. With the average monthly growth rate estimated at 1 cm [32], each 3 cm segment represents approximately three months of integrated cortisol secretion. This approach provides insights into cumulative hair cortisol levels three months before the MVC [11]. These assessments were conducted at the biochemical laboratory of Dresden University of Technology using liquid chromatography tandem mass spectrometry (LC-MS/MS), a highly accurate and reliable method for quantifying cortisol levels in biological samples according to the protocol by Gao et al. [33].

### Statistical analysis

The data analyses in the present study were performed using the *R* statistical software [34]. Due to a non-normal distribution of cortisol data, we used log-transformed values and executed the analyses using the *lmPerm* package in R. This method utilizes permutation tests for linear models. Unlike standard linear model functions, it adapts to cases where degrees of freedom for error are limited or nonexistent, such as in non-normal samples or the presence of outliers [34, 35] computing robust *p*-values. As an explanatory model effect size, we reported *R²*. To analyze the predictive value pre-MVC HCC_t1_ for psychological trauma sequelae 3 months post-MVC, we computed regression analyses. First, linear regressions were performed with trauma-related symptoms (PDS, IES-R subscales: intrusion, avoidance, hyperarousal) as dependent variables and pre-MVC HCC_t1_ as a predictor (see Table 1). To investigate the potential moderating effect of CT, we then calculated a regression model that included both pre-MVC HCC_t1_ and CTQ as individual predictors as well as their interaction term. Further, bivariate Pearson moment correlation were computed to consider possible HCC-confounders (e. g., sex, age, BMI, smoking, contraceptives; [11]). In case of significance, we rerun the regression analysis, respectively, with the identified confounding variable included as a covariate. In general, we applied a confidence level of 0.95 and a significance level of *p* < 0.05.

**Table 1:** Linear regression analysis of psychological symptoms following MVC based on pre-MVC HCC.

Outcome _t2_^+^	Predictor	*β*	*p*	*R² adj*.
PDS	HCC_t1_	0.14	0.034^*^	0.01
	HCC_t1_^a^	0.19	0.211	
	age	0.04	0.725	
IES-R total	HCC_t1_	0.20	0.030^*^	0.03
	HCC_t1_^a^	0.31	0.010^*^	
	age	−0.03	0.902	
IES-R intrusion	HCC_t1_^a^	0.21	0.029^*^	0.03
	HCC_t1_^a^	0.31	0.007^**^	
	age	−0.08	0.902	
IES-R avoidance	HCC_t1_	0.12	0.201	0.00
	HCC_t1_^a^	0.17	0.458	
	age	−0.003	0.980	
IES-R hyperarousal	HCC_t1_	0.20	0.023^*^	0.03
	HCC_t1_^a^	0.31	0.006^**^	
	age	−0.08	0.803	

*PDS* posttraumatic diagnostic scale, Pre-MVC->HCC_t1_ Hair segment before the accident (0–3 Months), *IES-R* impact of event scale – Revised.

^a^after controlling for age. Outcome (t2)^+^ symptom measurement post-MVC.

^*^p < 0.05, ^**^p < 0.01.

## Results

### Sample characteristics

The total sample comprised *N* = 272 participants (*n* = 164 females, *n* = 108 men). The majority of participants were German (97.6%) and possessed either a university degree or apprenticeship qualification. The mean age was *M* = 42.15 years (*SD* = 13.92). Five participants exhibited a HCC value < 1 and were excluded from the analyses. Additional sample characteristics are presented in Table 2. Concerning the characteristics of the psychological and biological parameters, the participants exhibited similar symptom severity at both measurement timepoints (see Table 3).

**Table 2:** Associations between sociodemographic aspects and HCC before the MVC.

HCC_t1_		
Sex		
Sex, females *n*(%)	164 (60.7)	−0.03
Age in yrs., *M (SD)*	42.15 (13.92)	0.18^*^
BMI in kg/m², *M(SD)*	18.98 (12.55)	0.04
Smokers, *n*(%)	121 (45)	0.04
Contraceptives	56 (25)	0.09
Education^a^		
- High school diploma	101 (46.5)	
- 10th grade	99 (45.6)	
- 8th grade	16 (4.8)	
CTQ		
- Mild	81 (39.7)	
- Moderate	63 (30.9)	
- Severe	60 (29.4)	

*CTQ* childhood trauma questionnaire: Trauma severity is categorized as follows: 25–34 (mild), 35–39 (moderate), and 40–125 (severe). HCC_t1_ hair segment before the accident (0–3 Months).

^a^In the German education system, a high school diploma qualifies for university admission. While the diploma until the 8–10th grade qualifies for vocational training. *Yrs* years.

^*^p ≤ 0.05.

**Table 3:** Psychological and biological characteristics of the sample (*N* = 272).

	M	SD	Differences over time^a^	df	t	p	d [95%-CI]
PDS _t1_	10.11	9.95	PDS _t1–t2_	472.86	0.66	0.512	0.06 [−0.12, 0.24]
PDS _t2_	9.52	9.56					
IES-total _t1_	23.89	20.85	IES-total _t1–t2_	220.64	0.324	0.746	−0.04 [−0.26, 0.19]
IES-total _t2_	24.72	23.03					
IES-Intrusion _t1_	9.31	8.19	IES-Intrusion _t1–t2_	225.34	1.19	0.237	0.14 [−0.09, 0.37]
IES-Intrusion _t2_	8.19	7.81					
IES-Avoidance _t1_	8.53	7.87	IES- Avoidance _t1–t2_	238.30	0.79	0.431	0.09 [−0.14, 0.32]
IES-Avoidance _t2_	7.82	7.74					
IES-Hyperarousal _t1_	6.14	6.57	IES- Hyperarousal _t1–t2_	224.26	0.44	0.664	0.05 [−0.18, 0.28]
IES-Hyperarousal _t2_	5.80	7.14					
CTQ	37.14	6.60					
HCC_t1_	5.31	5.15					

*PDS* posttraumatic disorder scale, *PDS*-reference values, 0 No symptoms, 1–10 mild symptoms, 11–20 moderate symptoms, 21–35 moderate to severe symptoms. 36 severe symptoms. IES-R-Total above 33 PTSD-diagnosis. *HCC* hair cortisol concentration (pg/mg), *HCC*_*t1*_ hair segment before the accident (0–3 Months).

^a^t-test values are reported.

#### Confounders

There were no significant correlations between HCC_t1_ and sex (*r* (251) = −0.05, *p* = 0.921), BMI (*r* (226) = 0.04, *p* = 0.570), smoking (*r* (250) = 0.04, *p* = 0.340), as well as contraceptives (*r* (209) = 0.09, *p* = 0.070). Only age (*r* (146) was correlated to HCC_t1_ = 0.19, *p* = 0.02. Its impact on the analyses is outlined below.

### Regression analyses

To test our hypothesis, first we examined (see Table 1) whether pre-MVC HCC_t1_ predicts psychological symptoms related to PTSD (i.e., PDS, avoidance, intrusion, hyperarousal) at three months post-MVC. The results revealed a significant prediction of trauma related symptoms except for IES-Avoidance (see Table 1). The results changed after adjustment of age:

1. **PDS**: A significant positive prediction was found between Pre-MVC HCC_t1_ and PDS scores at three months post-MVC (*β* = 0.14, *p* = 0.034), accounting for 1% of the variance in PDS symptoms. However, after controlling for age, this prediction was not significant suggesting that the effect may be influenced by age-related factors.
2. **IES-R** Total Score: Pre-MVC HCC_t1_ significantly predicted IES-R total score at three months post-MVC (*β* = 0.20, *p* = 0.030), explaining 3% of the variance in overall PTSD symptoms. This prediction remained significant after adjusting for age.
3. **IES-R Intrusion Subscale:** Pre-MVC HCC_t1_ significantly predicted symptoms of intrusion both before (*β* = 0.21, *p* = 0.029) and after controlling for age (*β* = 0.31, *p* = 0.007), indicating that higher initial cortisol levels predicted greater intrusion symptoms at three months post-MVC.
4. **IES-R Avoidance Subscale:** No significant prediction was found or after controlling for age suggesting that initial cortisol levels did not influence the avoidance response.
5. **IES-R Hyperarousal Subscale**: Pre-MVC HCC_t1_ significantly predicted hyperarousal symptoms (*β* = 0.20, *p* = 0.023). This relationship strengthened after controlling for age (*β* = 0.31, *p* = 0.006), indicating a robust association between early post-MVC cortisol levels and later hyperarousal symptoms.

In summary, these results indicate that higher premorbid HCC are predictive of greater PTSD symptoms, particularly in the domains of intrusion and hyperarousal, three months post-MVC. The predictions between premorbid HCC and these symptoms remained significant even after controlling for age, highlighting the potential role of cortisol as a biomarker for persistent stress responses following trauma exposure.

### Moderation analysis

In investigating the moderating influence of CTQ on the interplay between HCC and PTSD related symptoms (i.e., PDS, avoidance, intrusion, hyperarousal) we employed multiple regression models. The results indicated overall a significant model fit for every calculation. However, the results change after adjustment for age (see Table 4). The results changed as follows after controlling for age.

1. **PDS**: The moderation model was significant both before (*F* = 6.78, *p* < 0.001) and after controlling for age (*F* = 6.45, *p* < 0.001, R²_adj = 0.15) explaining 15% of the variance in PTSD symptoms. Specifically, CTQ was a significant predictor of PDS before (*β* = 0.29, *p* < 0.001) and after controlling for age (*β* = 0.36, *p* < 0.001). However, neither pre-MVC HCC_t1_ nor the interaction term (pre-MVC HCC_t1_ × CTQ) were significant predictors in either model (*p* > 0.05), suggesting that while CT is a robust predictor of later PTSD symptoms, it does not significantly moderate the relationship between HCC levels and PTSD symptoms.
2. **IES-R Total Score**: The moderation model was significant initially (*F* = 2.89, *p* < 0.001) but lost significance after controlling for age (*F* = 2.44, *p* = 0.072). CTQ was not a significant predictor of IES-R total scores (p > 0.05) in either model. However, pre-MVC HCC_t1_ was a significant predictor both before (*β* = 0.28, *p* = 0.002) and after controlling for age (*β* = 0.30, *p* = 0.015). The interaction term was, however not significant, indicating no moderation effect of CT on the relationship between HCC and overall PTSD symptoms.
3. **IES-R Intrusion Subscale:** The moderation model was significant before (*F* = 2.93, *p* = 0.038) but became marginally non-significant after controlling for age. CTQ was a significant predictor of intrusion symptoms both before (*β* = 0.17, *p* = 0.019) and after controlling for age (*β* = 0.23, *p* = 0.014). pre-MVC HCC_t1_ was also a significant predictor before (*β* = 0.16, *p* = 0.048) but became marginally non-significant after controlling for age (*β* = 0.16, *p* = 0.054). The interaction term was not significant in either model, indicating no moderating effect of CT on intrusion symptoms.
4. **IES-R Avoidance Subscale:** The moderation model was significant before (*F* = 2.97, *p* = 0.036) but became marginally non-significant after controlling for age (*F* = 2.14, *p* = 0.075). CTQ was a significant predictor before controlling for age and remained significant afterward (*β* = 0.23, *p* = 0.014). pre-MVC HCC_t1_ was not a significant predictor in either model and the interaction term were non-significant suggesting no moderating effect of CT on avoidance symptoms.
5. **IES-R Hyperarousal Subscale:** The moderation model was significant before controlling for age (*F* = 2.93, *p* = 0.038) but became marginally non-significant after controlling for age. Pre-MVC HCC_t1_ was a significant predictor both before and after controlling for age (*β* = 0.16, *p* = 0.042). The interaction term was not significant in either model indicating no moderating effect of CT on hyperarousal symptoms.

**Table 4:** Moderation analysis of childhood trauma on the relationship between PTSD symptoms and pre-MVC HCC.

Outcome _t2_	Predictor	*F*	*β*	*p*	*R² adj*.	*F*	*β*	*p*	*R² adj*.
	Moderation model^a^					Model^b^			
PDS		6.78		<0.001	0.08	6.45		<0.001	0.15
	CTQ		0.29	<0.001			0.36	<0.001	
	HCC_t1_		0.07	0.902			0.35	0.526	
	HCC_t1_*CTQ		0.05	0.332			0.05	0.784	
						age	−0.05	0.745	
IES-R total									
		2.89		<0.001	0.08	2.44		0.072	0.05
	CTQ		0.12	0.095			0.11	0.122	
	HCC_t1_		0.28	0.002			0.30	0.015	
	HCC_t1_*CTQ		−0.02	0.921			−0.02	0.803	
						age	−0.07	0.426	
IES-R intrusion									
		2.93		0.038	0.06	2.14		0.075	0.05
	CTQ		0.17	0.019			0.23	0.014	
	HCC_t1_		0.16	0.048			0.16	0.054	
	HCC_t1_*CTQ		−0.05	0.998			−0.07	0.784	
						age	−0.05	0.705	
IES-R avoidance									
		2.97		0.036	0.06	2.14		0.075	0.05
	CTQ		0.24	0.048			0.23	0.014	
	HCC_t1_		0.16	0.116			0.16	0.058	
	HCC_t1_*CTQ		−0.06	0.823			−0.07	0.784	
						age	−0.05	0.705	
IES-R hyperarousal									
		2.93		0.038	0.06	2.14		0.082	0.05
	CTQ		0.17	0.686			0.23	0.061	
	HCC_t1_		0.16	0.046			0.16	0.042	
	HCC_t1_*CTQ		−0.05	0.989			−0.07	0.225	
						age	−0.05	0.666	

*a* without controlling for age, *b* after controlling for age, *CTQ* childhood trauma questionnaire, Pre-MVC->HCC_t1_ hair segment before the accident (0-3 Months), *PDS* posttraumatic diagnostic scale, *IES-R* impact of event scale – Revised.

## Discussion

This study extends previous findings by investigating the predictive value of pre-MVC hair HCC for PTSD symptoms and examining whether CT moderates this relationship following adversity in participants without a history of CT. Overall, the presented findings demonstrated that CT is a consistent and significant predictor of trauma-related psychological symptoms, including specific PTSD responses (i.e., intrusion, avoidance). Importantly, this effect persists even after adjustment of age, indicating that the impact of early trauma on adult mental health is robust across different age groups. On the other hand, while premorbid HCC was found to be a significant predictor of total PTSD symptoms, as well as specific domains like intrusion and hyperarousal, the interaction between pre-MVC HCC_t1_ and CT was not significant in any model. This suggests that the influence of premorbid HCC on psychological outcomes is independent of CT history and age does not alter this relationship.

The study at hand confirmed that elevated pre-MVC HCC is predictive of subsequent PTSD symptoms and extended this association to intrusion and hyperarousal symptoms. However, contrary to our expectations, CT did not reveal a moderation effect. Instead, CT emerged as an independent predictor of PTSD symptoms, underscoring its pivotal role in symptom manifestation. In detail, our analyses revealed that higher **pre-MVC HCC** predicted subsequent symptoms of PTSD including intrusion and hyperarousal following adversity. These findings extend those of Petrowski et al., [20] who observed elevated integrated HCC prior MVC as predictive of only avoidance behavior. Moreover, our results validate those of Pacella et al. [19] who revealed that higher HCC (i.e., peri-traumatic) predicted significant increases in overall PTSD at follow-up (within 60 days post-injury). On the other hand, these findings contradict previous association between pre-traumatic decreased HCC and an increased risk for symptoms of PTSD [18]. This discrepancy can be explained by differences in time of data collection and sample population. Steudte-Schmiedgen et al. [18] found a trend for lower integrated HCC prior-trauma and PTSD symptoms in traumatized soldiers. The latter might by indicative of frequent traumatization in the past [36], while our participants reflect a civilian sample with subclinical PTSD symptoms prior- and post-MVC. A further explanation draws from an integrative model [4, 17], which elucidated that sustained elevation of cortisol levels leads to hypersensitization of the negative feedback loop of the HPA axis, ultimately leading to a decrease in long-term basal cortisol secretion. These mechanisms have been proposed to play a role in increasing susceptibility to PTSD symptoms among vulnerable individuals [15]. Finally, contrary to previous findings [8–10] the present study did not identify CT as a moderator in the relationship between pre-MVC HCC and PTSD symptoms. This discrepancy may stem from methodological differences, such as the failure to account for recent adversity in prior studies. Additionally, our findings demonstrated small effect sizes, being potentially underpowered. Nonetheless, our results consistently demonstrated that CT, when compared to integrated pre-MVC HCC levels, independently predicted symptoms of PTSD post-MVC. These results emphasize that while both CT and pre-MVC HCC independently affect trauma responses in adulthood, they do not interact in a way that would indicate CT moderates its link. This finding validates previous research regarding the enduring influence of adversity on the development of trauma-related psychopathology [7, 37]. Clinical implications emphasize integrating PTSD interventions that address both biological factors (e.g., cortisol levels) and the lasting psychological impact of early trauma, treating them as distinct yet complementary therapeutic targets.

In sum, our main findings extended previous evidence by showing that elevated long-term HCC before adversity can be considered as risk factor for PTSD symptoms. Further our data supported past results on the enduring influence of early-adversity on mental health amidst challenging conditions [6]. In the present study, several factors must be considered to contextualize our findings. Firstly, we contacted participants within 10 days post-MVC, which deviated from the 7-day interval specified by the self-report scales (e.g., IES-R). Consequently, the reported PTSD symptoms may reflect a peri-post traumatic period rather than exclusively the pre-MVC timeframe. Additionally, our analysis of cortisol data did not account for variables such as hair washing frequency, batch differences, storage conditions, or circadian effects (e.g., time of day when the MVC occurred; [38]), which could act as confounding factors. Furthermore, our study experienced a dropout rate of 13.13%, which, although consistent with similar past studies [39, 40], poses constraints on our results. This dropout was primarily due to changes in contact information at follow-up and the high proportion of MVC victims transported to specialized trauma clinics. Lastly, out of the full sample of *N* = 272, a total of *n* = 7 individuals in the analysis did not report their age. Thus, there is a potential influence of missingness on the statistical power of the analysis. Potentially, the power of the models decreases due to the missing values for age and as a consequence the significance of the reported results also slightly decreases after including age.

Despite these limitations, our study boasts several strengths, including a rigorous design, comprehensive data collection, and a targeted population. Although the validity of HCC as a historical biomarker has been questioned in animal studies [41], it has been robustly validated in human research against cumulative cortisol levels measured through saliva and urine, demonstrating strong retest reliability under stable environmental conditions [11, 42]. Thus, our data reflects long-term endocrine cortisol secretion, providing a retrospective and objective measure of chronic stress. Compared to other studies are HCC samples were collected shortly after the MVC. Furthermore, our study focuses on a well-defined cohort of individuals with TBI resulting from MVCs, a group highly relevant to PTSD research. We employed stringent exclusion criteria, including the elimination of several confounding factors (e.g., chronic illnesses, psychopharmaceutical use, and shift work) facilitating an accurate assessment. Our findings align with theoretical frameworks on stress, offering further evidence for the predictive utility of integrated HCC (e.g., [43]). To provide a clearer picture of these preliminary results, future studies should consider analyzing the effects of other confounders, as well as the time of day at which the MVC occurred. Additionally, future research could benefit from investigating factors related to resilience or post-traumatic growth that may protect individuals from developing trauma sequelae.

## Conclusion

Our study demonstrates that elevated pre-MVC HCC predicts PTSD symptoms, highlighting the role of chronic stress and HPA axis dysregulation in posttraumatic psychopathology. CT did not moderate this relationship, suggesting that HCC’s impact on PTSD is independent of early adverse experiences. However, CT emerged as an independent predictor of PTSD at the 3-month follow-up, underscoring its lasting influence on psychological trauma vulnerability. Clinically, findings emphasize that interventions addressing PTSD should consider both biological (systemic cortisol) and the long-term effects of early trauma on mental health, but may treat them as parallel rather than interdependent processes (e. g., combining psychotherapy with endocrinological evaluation and stress management).

## Data Availability

The datasets used and/or analyzed during the current study are available from Prof. Katja Petrowski: kpetrows@uni-mainz.de on reasonable request.
